# Sarcopenic obesity is attenuated by E-syt1 inhibition via improving skeletal muscle mitochondrial function

**DOI:** 10.1016/j.redox.2024.103467

**Published:** 2024-12-12

**Authors:** Chao Song, Wu Zheng, Guoming Liu, Yiyang Xu, Zhibo Deng, Yu Xiu, Rongsheng Zhang, Linhai Yang, Yifei Zhang, Guoyu Yu, Yibin Su, Jun Luo, Bingwei He, Jie Xu, Hanhao Dai

**Affiliations:** a Department of Orthopedics, Shengli Clinical Medical College of Fujian Medical University, Fujian Provincial Hospital, Fuzhou University Affiliated Provincial Hospital, School of Medicine, Fuzhou University, Fuzhou, 350001, China; b School of Mechanical Engineering and Automation, Fuzhou University, Fuzhou, 350001, China; c Department of Traditional Chinese Medicine, Fujian University of Traditional Chinese Medicine, Fuzhou, 350122, China; d Department of Pediatrics, First Affiliated Hospital of Fujian Medical University, Fuzhou, 350005, China

**Keywords:** Sarcopenic obesity, E-syt1, Myogenesis, Mitochondria, Mitophagy

## Abstract

In aging and metabolic disease, sarcopenic obesity (SO) correlates with intramuscular adipose tissue (IMAT). Using bioinformatics analysis, we found a potential target protein Extended Synaptotagmin 1 (E-syt1) in SO. To investigate the regulatory role of E-syt1 in muscle metabolism, we performed in vivo and in vitro experiments through E-syt1 loss- and gain-of-function on muscle physiology. When E-syt1 is overexpressed in vitro, myoblast proliferation, differentiation, mitochondrial respiration, biogenesis, and mitochondrial dynamics are impaired, which were alleviated by the silence of E-syt1. Furthermore, overexpression of E-syt1 inhibited mitophagic flux. Mechanistically, E-syt1 overexpression leads to mitochondrial calcium overload and mitochondrial ROS burst, inhibits the fusion of mitophagosomes with lysosomes, and impedes the acidification of lysosomes. Animal experiments demonstrated the inhibition of E-syt1 increased the capacity of endurance exercise, muscle mass, mitochondrial function, and oxidative capacity of the muscle fibers in OVX mice. These findings establish E-syt1 as a novel contributor to the pathogenesis of skeletal muscle metabolic disorders in SO. Consequently, targeting E-syt1-induced dysfunction may serve as a viable strategy for attenuating SO.

## Abbreviations

SO: Sarcopenic obesity
IMAT: Intramuscular adipose tissue
E-syt1: Extended Synaptotagmin 1
WAT: White adipose tissue
DMD: Duchenne muscular dystrophy
ER: Endoplasmic reticulum
FBS: Fetal bovine serum
PBS: Phosphate-buffered saline
qRT-PCR: Real-time quantitative PCR
TEM: Transmission electron microscopy
CCK-8: Cell counting kit-8
EDU: 5-ethynyl-2′-deoxyuridine
CS: Citrate synthase
mtDNA: mitochondrial DNA
TMRE: Tetramethylrhodamine ethyl ester
MTR: Mito-Tracker Red
FF: Form factor
AR: Aspect ratio
OVX: Ovariectomy
AAV9: Adeno-associated virus 9
GA: Gastrocnemius
SDH: Succinate dehydrogenase
SD: Standard deviation
EMCS: ER-mitochondria contact sites
nDNA: Nuclear DNA
MMP: Mitochondrial membrane potential

## 1 Introduction

Sarcopenia, a common ailment among older individuals, is characterized by a progressive reduction in muscle mass and functionality. This disease has been linked to different negative health consequences, such as increased vulnerability to fractures, decline in functionality, and death [1]. Observation of intramuscular adipose tissue (IMAT) has been reported in certain pathological conditions, including Duchenne muscular dystrophy (DMD), sarcopenia, diabetes, and obesity [2]. Sarcopenic obesity (SO) refers to a clinical condition where both sarcopenia and obesity coexist. The estimated prevalence of SO ranges from 5 % to 10 %, with a higher occurrence observed among individuals aged 80 years or older [[3], [4], [5]]. Moreover, the prevalence of SO tends to rise with advancing age [5]. A recent study pointed out that sarcopenia can occur concurrently with, and may be worsened by, body fat gain in the context of obesity [6]. The etiology of SO is multifaceted, involving changes in body composition related to age (increase in visceral fat and IMAT), chronic inflammation throughout the body, insulin resistance, and lifestyle changes [5]. Many adipokines play key roles in muscle function, leptin and adiponectin demonstrate a strong correlation with sarcopenia. Administering leptin intravenously to adult male rats led to a significant enhancement in the size of muscle fibers, indicating a possible correlation with the stimulation of insulin signaling pathways [7]. Nevertheless, it has been noted that increased concentrations of leptin in aged (48 weeks) female rats are linked to ectopic muscular inflammation, potentially resulting in the deterioration of muscle tissue [8]. Mice lacking adiponectin display a phenotype marked by obesity and reduced sensitivity to insulin. In contrast, introducing adiponectin as a transgene exclusively into skeletal muscle enhances insulin sensitivity [9].

Extended Synaptotagmin 1 (E-syt1) is a protein found in the endoplasmic reticulum (ER) that facilitates the tethering and transportation of lipids in a manner dependent on the concentrations of Ca^2+^ [10]. A recent study revealed that E-syt1 is extensively dispersed in the cytoplasm, with a portion localized at endoplasmic reticulum-mitochondria contact sites (EMCS) for mitochondrial lipid transport and respiration [11]. Pan et al. showed that ANKRD22-expressing 293T cells exhibited enlargement and vacuole-like alterations in their mitochondria, along with a reduction in mitochondrial cristae, following the overexpression of E-syt1. Additionally, E-syt1 was identified as a crucial molecule involved in the accumulation of lipids and the degeneration of mitochondria [12]. Jun et al. observed that their invasiveness decreased when E-syt1 expression was eliminated in CD74-ROS-expressing cells. Furthermore, the researchers identified the phosphorylation of E-syt1 on tyrosine sites in various malignancies [13]. TFE3 facilitates the increase of E-syt1 and the creation of a heterodimer with Syt7, a protein found in the lysosomal membrane. This process leads to ER fragmentation, the release of calcium ions, and lysosomal exocytosis [14]. Furthermore, previous research has indicated that the expression of E-syt1 is elevated during the differentiation of adipocytes in 3T3-L1 cells, and it also influences the transport of glucose in 3T3-L1 adipocytes [15].

Mitochondria play a crucial role in preserving metabolic equilibrium within skeletal muscle, the most metabolically active tissue undergoing extensive remodeling in response to myriad physiologic or pathophysiological stimuli. The regulation of mitochondrial biogenesis and mitophagy is of utmost importance for maintaining the quantity and functionality of mitochondria and cellular homeostasis and adaptations to metabolic requirements [16]. Mitophagy, fission/fusion, mitochondrial biogenesis, and cellular energy metabolism work together to maintain mitochondrial function [[16], [17], [18]]. Maintaining optimal mitochondrial health is essential for muscle tissue's survival and proper operation. Mitophagy, a specific type of autophagy, is vital in monitoring and removing damaged and harmful mitochondria. It has gained significant attention in research [19]. It is worth mentioning that there has been an observed decrease in autophagy levels in older muscles, which could result in reduced mitophagy and, therefore, indicate the existence of mitochondrial dysfunction [17,[20], [21], [22]]. Various mitophagy pathways have traditionally been identified, like the Pink1/Parkin pathway, the Bnip3/Nix pathway, and the Fundc1 pathway [23]. The present investigation revealed that E-syt1 demonstrates elevated expression levels in SO muscle tissue. However, the precise physiological and pathological roles of E-syt1 in skeletal muscle have yet to be investigated. Consequently, we conducted investigations to assess the impact of E-syt1 on skeletal muscle mitochondrial homeostasis, both in vivo and in vitro, and to elucidate the underlying molecular mechanism.

## 2 Materials and methods

### 2.1 Animals

The Animal Experiment Center of Fujian Medical University supplied five 4-month-old C57/BL6 mice, 48 12-week-old female C57/BL6 mice, and five 24-month-old C57/BL6 mice. The animals were fed a standard diet, housed 5 per cage under a designated room, following a 12-h light-dark cycle, and provided filtered water. The Animal Ethics Committee of Fujian Provincial Hospital approved all animal experiments (IACUC-FPH-SL-20230724[0029]). The animal experiments followed the ARRIVE guidelines (https://www.nc3rs.org.uk/arrive-guidelines) and the National Research Council's Guide for the Care and Use of Laboratory Animals.

### 2.2 Cell culture and differentiation

Mouse C2C12 myoblast cells were purchased from the cell bank of the Chinese Academy of Sciences (Cat#GNM26, Shanghai, China). C2C12 cells were cultured in DMEM with the addition of 10 % fetal bovine serum (FBS) (Cat#16000044, Gibco, USA). All cells were cultured with 1 % penicillin/streptomycin (Cat#1541, Gibco, USA) at 37 °C in 5 % CO_2_. Mycoplasma contamination was not detected in each cell line before use. To stimulate C2C12 myoblast differentiation, cells were switched into a differentiation medium (DMEM medium supplemented with 2 % horse serum and 1 % penicillin/streptomycin) once reaching 80–90 % confluence. The myogenic differentiation assay was conducted over seven days.

### 2.3 Bodipy staining

Bodipy staining of skeletal muscle was done as previously described [24]. The frozen slides were fixed by immersion in ice-cold 4 % paraformaldehyde for 10 min. Then, the slides were washed three times in deionized water and incubated for 30 min with the lipophilic dye Bodipy 493/503 (Cat#790389, Sigma, USA) at 37 °C. Digital photographs were captured from each section under fluorescence illumination, and the area content of the lipid droplet was quantified using ImageJ software. Digital pictures were taken from each section under fluorescence illumination, and the adipocyte area content was quantified using ImageJ software.

### 2.4 Real-time quantitative PCR (qRT-PCR)

Cells were subjected to RNA extraction using TRIzol Reagent (Cat#15596026, Invitrogen, USA), followed by cDNA synthesis using a cDNA synthesis kit (Cat#RR047Q, Takara, Japan). Real-time PCR was performed with TB Green® Premix Ex Taq™ II (Cat#RR820A, Takara, Japan) and StepOnePlus Real-Time PCR System. GAPDH was utilized as an internal control to normalize the data. Primers used for qPCR are listed in Table S1. Gene expression was quantified by using the 2^−ΔΔCt^ method.

### 2.5 Transmission electron microscopy (TEM)

We examined the ultrastructural features and composition of mitochondria in the C2C12 cells or gastrocnemius muscle using TEM as described previously [25,26]. Briefly, cells or tissue were preserved in a solution containing 2 % glutaraldehyde (Cat#16220, EMS, USA) and 2 % paraformaldehyde (Cat#15710, EMS, USA) in PBS buffer. Subsequently, they were further treated with 1 % osmium tetroxide (Cat#19110, EMS, USA) for fixation. To prepare thin sections, a solution containing 2 % uranyl acetate (Cat#22400-2, EMS, USA) and 0.2 % lead citrate (Cat#17810, EMS, USA) was used for staining and subsequently observed using a 100 kV electron microscope (Cat#HT-7700, Hitachi, Japan). The Image J software was used to quantify the ultrastructure of mitochondria and mitophagosomes (when a mitochondrion or cristae remnants were observed inside double-membrane structures). The quantification of mitochondrial distribution (number per μm^2^) and density (μm^2^ x number per μm^2^ x 100) was conducted by manually outlining and counting mitochondria in a double-blinded manner. A total number of abnormal mitochondria (i.e., mitochondria with disrupted membranes, loss of cristae, and vacuolization) were also assessed as previously described [26]. The number of mitophagosomes per 100 μm^2^ was counted.

### 2.6 Cell counting kit-8 (CCK-8) assay

Cell proliferation was assessed using CCK-8 staining (Cat#C0038, Dojindo, Japan). A total of 1000 C2C12 cells were seeded in 96-well plates. A 10 μl CCK-8 solution was added and left to incubate at 37 °C for 1 h. The microplate reader (SpectraMax iD5, MD, USA) was used to measure the absorbance. The CCK-8 assay was performed 24 h, 48 h,72 h, and 96 h.

### 2.7 5-ethynyl-2′-deoxyuridine (EDU) cell proliferation assay

The 10 μM EDU medium was prepared per the guidelines provided by the manufacturer (Cat#C10310-3, Cat#C10310-1, RiboBio, China). The C2C12 cells were seeded into the 24-well plate. After the C2C12 cells reached the appropriate confluence, the medium was replaced with 100 μL of EDU medium and incubated at 37 °C in 5 % CO_2_ for 2 h. Subsequently, the C2C12 cells were fixed in 4 % paraformaldehyde for 20 min and subjected to a 30-min incubation with Apollo® reagent (100 μL) at room temperature. Afterward, the cells were stained with Hoechst dye for 30 min and examined using a fluorescence microscope. The ratio of EdU-positive cells to the total number of Hoechst-positive cells was calculated to determine cell proliferation.

### 2.8 Analysis of the cell cycle

The cell cycle was detected using the cell cycle detection kit (Cat#KGA512, Keygen Biotech, China). The cells were synchronized by serum starvation for 24 h and induced to re-enter the cell cycle by an exchange of 10 % fetal bovine serum for 24 h. The synchronized cells were harvested and fixed in 70 % ethanol at 4 °C for 12 h. After that, the cells were washed twice with cold PBS and stained with propidium iodide (PI)/RNase A solution at 37 °C for 30 min without light. The cell cycle was measured by flow cytometry. Using the ModFit software, the FACS verse (Accuri C6 Plus, BD, USA) was employed to test the samples and determine the percentage of cells in each cell cycle stage.

### 2.9 Lentivirus construction

Lentivirus construction of E-syt1-shRNA, control shRNA, E-syt1-overexpression, and control-overexpression were purchased from Genechem Company (Shanghai, China).

The sequences of two E-syt1-targeting shRNAs and non-target control shRNA are listed in Table S2. 5 × 10^4^ cells were plated on 12-well plates; cells at 30–50 % confluence were transfected using HitransG Transfection Reagent P (Cat#REVG005, Genechem, China) by the manufacturer's instructions. The transfected cells were selected with 4 μg/ml puromycin (Cat#ST551-10 mg, Beyotime, China) for 10 days to establish stably expressing cells. qRT-PCR and western blotting evaluated the transfection effectiveness. Silencing and overexpression C2C12 cells were transiently transfected with pMRX-IP-GFP-LC3-RFP reporter (Cat#84573, Addgene, China) to analyze autophagic flux.

### 2.10 Isolation of mitochondria

According to the manufacturer's protocols, mitochondrial proteins were extracted using a Mitochondria Protein Extraction Kit (Cat#C3601, Beyotime, China). Briefly, cells were dispersed and centrifuged to obtain cell pellets. Then, they were resuspended with 1 mL lysis buffer and transferred into a glass grinder for homogenization. The homogenate was centrifuged at 800g for 5 min at 4 °C. The supernatant was collected and then centrifuged at 15,000g for 10 min at 4 °C, followed by giving up the medium buffer. Finally, a lysis buffer containing a protease inhibitor was used to resuspend the pellet to obtain the mitochondrial protein.

### 2.11 Western blotting analysis

The protein was abstracted via RIPA lysis buffering solution to which phosphatase and protease (Cat#KGP2100, Keygen Biotech, China) inhibitors were added. Proteins underwent electrophoresis on SDS-PAGE gels with a concentration of 12.5 % or 10 % (w/v), then transferred onto 0.2 μm or 0.45 μm PVDF membrane (Cat#88520, Cat#88585, Thermo, USA). Membranes were blocked with 5 % non-fat dry milk in Tris-buffered saline containing 0.1 % Tween 20 (TBST) for 1 h at room temperature. Each blot was incubated with its primary antibody diluted in TBST at 4 °C overnight. Afterward, they were incubated with appropriate secondary antibodies at 37 °C for 1 h. Subsequently, blots were visualized using the BeyoECL Plus Chemiluminescence Kit (Cat#P0018S, Beyotime, China) and an automatic digital gel/chemiluminescence image analysis system. Antibodies are listed in Table S3.

### 2.12 Immunohistochemistry/immunofluorescence staining

Mouse gastrocnemius muscle samples were used for immunohistochemistry as described previously [27]. After deparaffinization and dehydration of paraffin-embedded gastrocnemius muscle, 10 μm-thick sections were incubated with primary antibody overnight at 4 °C. Then, sections were incubated with secondary antibodies followed by the DAB (3,3′- diaminobenzidine) horseradish peroxidase color development kit (Cat#P0202, Beyotime, China), and nuclei were counterstained with hematoxylin. The images were obtained using the microscope (Leica, Wetzlar, Germany) and quantified by ImageJ. The fixed cells and sections were blocked with a quick-lock blocking buffer for immune staining (Cat#P0260, Beyotime, China) for 15 min at room temperature to perform immunofluorescence staining. After that, they were exposed to primary antibodies and incubated overnight at 4 °C. Next, the cells and sections were stained with secondary antibodies appropriately labeled with fluorescence for 2 h at room temperature. Then, DAPI staining was performed for 5 min and finally visualized using a fluorescence microscope.

### 2.13 Oxygen consumption measurement

The Seahorse XF24 extracellular flux analyzer (Seahorse Bioscience, Agilent Technologies, USA) was used to assess the oxygen consumption rate (OCR) in mitochondria following manufacturer-recommended protocols. On the day before assay, 30,000 transfected cells were placed in each well of microplates, and a sensor cartridge was hydrated overnight in XF Calibrant at 37 °C in a CO_2_-free incubator. The next day, the cells were rinsed and incubated with XF-Base Medium (non-buffered DMEM containing 10 mM glucose, 2 mM glutamine, and 1 mM pyruvate, pH 7.4) for 1 h in an incubator without CO_2_. To measure OCR, oligomycin (1.5 μM), FCCP (0.5 μM), and rotenone/antimycin A (0.5 μM) were successively added to every well. After the seahorse measurements, OCR was normalized to citrate synthase (CS) activity.

### 2.14 Citrate synthase activity

CS activity was evaluated according to the procedure described by Zhang et al. and Lee et al. [28,29]. After the seahorse assay, any remaining medium was removed. CS reaction buffer (200 mM Tris buffer at pH 8.0 (Cat#T6664, Sigma, USA), 0.2 % Triton X-100 (v/v) (Cat#9002931, Sigma, USA), 10 μM DTNB (Cat#D8130, Sigma, USA), 1 mM Acetyl-CoA (Cat#A2181, Sigma, USA)) was added to each well. Subsequently, each well was introduced 10 mM oxaloacetate (Cat#O4126, Sigma, USA). The microplate reader (SpectraMax iD5, MD, USA) was used to measure the absorbance at 412 nm [28,29].

### 2.15 Measurement of mitochondrial DNA (mtDNA) copy number

The mtDNA copy number detection was performed to determine the number of mitochondria [30]. According to the manufacturer's instructions, total cellular DNA was isolated using PureLink DNA Mini Kit (Cat#K182002, Invitrogen, USA). According to Malik et al., the mtDNA copy number was quantified by qPCR using primers for specified mitochondrial and nuclear genome targets (mMitoF1/R1 and mB2MF1/R1, respectively) [31]. Primer sequences are provided in Table S1.

### 2.16 Measuring mitochondrial membrane potential

The tetramethylrhodamine ethyl ester (TMRE) mitochondrial membrane potential kit (Cat#C2001S, Beyotime, China) was used. According to the manufacturer's instructions, cells were stained with TMRE at 37 °C in a 5 % CO_2_ incubator for 30 min [32]. They were washed thrice with preheated PBS at 37 °C, and nuclei were counterstained with DAPI for 5 min and then imaged with a fluorescence microscope.

### 2.17 Mito-Tracker Red (MTR) staining

According to the manufacturer's instructions, the mitochondria were stained with Mito-Tracker Red CMXRos (Cat#C1049B-50 μg, Beyotime, China). The cells were incubated for 30 min, and then mitochondrial images were taken using a Zeiss LSM 510 inverted confocal microscope. The morphological characteristics of mitochondria were quantitatively determined using the NIH‐Image J software plugin, and essential aspects of mitochondrial morphology were recorded [33]. The measurement of mitochondrial size was determined using the mean area and mean perimeter. The morphology of the mitochondria was characterized using the form factor (FF) and aspect ratio (AR) for the 2D analysis [33,34].

### 2.18 Measurement of mitochondrial calcium levels

The mitochondrial Ca^2+^ levels were measured using Rhod-2 AM (Cat#R1245MP, Invitrogen, USA). Briefly, a stock solution of Rhod-2 was dissolved in DMSO, and C2C12 cells were incubated with Rhod-2 AM at a concentration of 5 μM at 37 °C in the dark for 30 min. Cells were washed three times to remove excess or non-specific probes loaded in mitochondria. After de-esterifying the mitochondrial AM esters, the fluorescence intensities were assessed using a fluorescence confocal microscope. The images for quantification were analyzed using Image J software.

### 2.19 Mitochondrial superoxide detection

Mitochondrial ROS was identified via the fluorogenic stain MitoSOX Red (Cat#M36008, Invitrogen, USA), targeted to mitochondria in alive cells. In short, cells were incubated in 5 μM work solution for 30 min without light at 37 °C, washed three times in a warm medium, and the nuclei were counterstained with DAPI for 5 min before being evaluated under a confocal microscope. Image quantification was conducted with ImageJ.

### 2.20 Measurement of mitophagy using mt-Keima

According to the manufacturer's instructions, cells were transfected with a lentivirus overexpressing the pH-sensitive fluorescent protein mt-Keima (Genechem, Shanghai, China). mt-Keima is a mitochondria-targeted protein that emits green light (458 nm) at a neutral pH and red light in mitochondria engulfed in acid lysosomes (534 nm). The ratio (534/458 nm) of mt-Keima emission light was calculated to reflect mitophagy [25,35].

### 2.21 Lysotracker staining

According to the manufacturer's instructions, the lysosomes were labeled with Lyso-Tracker Green (Cat#C1047S, Beyotime, China), a highly sensitive pH probe targeting acidic organelles such as lysosomes. The Lyso-Tracker Green staining solution was incubated with the cells at 37 °C for 30 min, after which the staining solution was discarded. Fresh cell culture medium was added, and imaging was conducted using a laser confocal microscope.

### 2.22 Measurement of cathepsin activity

According to the manufacturer's protocol, lysosomal cathepsin B enzymatic activity was detected using the Magic Red Cathepsin B Assay Kit (Cat#937, Immunochemistry technologies, China). Cells were incubated with Magic Red staining solution for 60 min at 37 °C protected from light, followed by staining with DAPI for 5 min. Images were acquired using confocal microscopy. Image quantification was conducted with ImageJ.

### 2.23 Design of animal experiments

Without treatment, 5 4-month-old and 5 24-month-old male mice were sacrificed for sampling. Randomly (random number table method), 48 12-week-old female mice were separated into two groups: the Sham group (n = 8) and the ovariectomy (OVX) group (n = 40). OVX mice were randomly assigned to one of five groups (n = 8) eight weeks after surgery. Adeno-associated virus 9 (AAV9) vectors encoding AAV9-sh-Scramble (AAV9-eGFP-U6-sh-Scramble), AAV9-shE-syt1 (AAV9-eGFP-U6-shE-syt1), AAV9-EV (AAV9-eGFP-U6-EV), and AAV9-E-syt1 (AAV9-eGFP-U6-E-syt1) were constructed by the Genechem Company (Shanghai, China). The sequences of AAV9-shE-syt1 and AAV9-sh-Scramble are listed in Table S2. The mice underwent multi-point injection into the Gastrocnemius (GA) using a 20 μl dilution of the above-described AAV9 particles in PBS, resulting in a final concentration of 2 × 10^11^ vector genomes (vg)/GA. In the Sham and Control groups, 20ul PBS was injected multifocally into bilateral gastrocnemius. Four weeks following injection, all animals commenced behavioral experiments and training on a treadmill. Following the sacrifice and weighing of all animals, bilateral gastrocnemius muscles were obtained, sectioned, weighed, and subjected to histological analysis. Bioluminescence imaging was performed using an IVIS Lumina Imaging System.

### 2.24 Exercise tolerance test

Behavioral tests were carried out four weeks after the AAV9 injection. Exercise tolerance was performed as previously described for AAV9-treated mice and their controls [36]. Before the experimental test, mice underwent a two-day acclimation phase where they explored the treadmill (Treadmill TSE Systems, Germany) for 5 min at a speed of 0 cm/s, followed by 5 min at a slow speed of 10 cm/s. The next day, the mice were positioned on a treadmill that gradually accelerated every 2 min by 5 cm/s at a 13 % angle until they reached the point of exhaustion. Despite mechanical stimulation, if the animal's hindlimbs stay on the electric grid for more than 10 s, it meets the exhaustion criterion. After the protocol, the speed, distance, and time were automatically collected.

### 2.25 Grip strength

A grip strength meter (Cat#XR501, XinRuan, China) was used to measure hindlimb grip strength, as previously described by Huang et al. [37]. Briefly, the animal was supported by the scruff of its neck and firmly latched onto a pull bar with their hindlimbs. Once the animal grasped the bar, it was steadily drawn backward along a horizontal trajectory while its upright stance was upheld. The display recorded the maximum force exerted before the animal released the bar. Grip strength was quantified as the mean of three trials with 1–2 min of recovery between trials, repeated on three days.

### 2.26 Histology assessment

The body weight of mice was quantified, and subsequently, the gastrocnemius muscles were dissected and directly weighed following the sacrifice of the animals. Gastrocnemius muscle was stored at −80 °C until use. To perform H&E and Masson staining, the GA was fixed in 4 % paraformaldehyde for 24 h, enclosed in paraffin, and transformed into 5 μm sections. H&E staining and Masson staining were conducted using the H&E staining kit (Cat#C0105 M, Beyotime, China) and the Masson staining kit (Cat#KGMST-8004, Keygen Biotech, China), respectively. For succinate dehydrogenase (SDH) staining, gastrocnemius muscles were embedded in OCT compound and sectioned at 10 μm thickness. The SDH activities were estimated in muscle tissues using the SDH staining kit (Cat#G2000, Solarbio, China) following the manufacturer's instructions. All image analysis was performed using ImageJ processing software. H&E stained histological slides were used to assess the minimal Feret's diameter. A minimum of 2000 fibers was utilized for each condition and measurement. The minimum ferret diameter is the shortest distance between two parallel tangents at opposite edges of the muscle fiber. Image analyses were conducted using ImageJ with a custom-developed macro identifying muscle fibers [38]. Quantified muscle fiber type numbers using MyoView, a fully automated CSA quantification method for skeletal muscle images applicable to any muscle [39].

### 2.27 Statistical analysis

All data are expressed as means ± standard deviation (SD). The data were analyzed using GraphPad Prism (9.0, Graph Software, USA). One-way ANOVA with Tukey's multiple comparisons post hoc test for multiple comparisons was applied to determine the statistical significance between the three groups. In contrast, student t-tests determined statistical significance between the two groups. A parametric two-way ANOVA (corrected with Sidak's multiple comparisons test) was used for numerous comparisons involving distinct groups at various time points. A p-value less than 0.05 was considered statistically significant.

## 3 Results

### 3.1 E-syt1 hinders the proliferation and differentiation processes of C2C12 myoblasts

Since SO is a high-risk geriatric syndrome predominantly observed in an aging population and is closely related to adipose tissue [40]. We identified the significantly up-regulated protein in adipose tissue and aged skeletal muscle through bioinformatics analysis. We searched multiple databases, such as the adipokines database (http://www.diabesityprot.org/) [41], the exo-adipokines database in Exocarta (www.exocarta.org), up-regulated mRNA in adipogenic differentiation (GSE185484), and up-regulated proteins in aging muscle (ProteomeXchange with identifier PXD027464 and PXD027490) [42]. The intersection results of these four databases were visualized using Venn diagrams. We identified three candidate proteins (E-syt1, Adipoq, and Ivd) (Fig. 1A). Based on the original young-aging muscle proteomics data (PXD027464 and PXD027490), we drew a heat map (Fig. 1B) and a volcano map (Fig. 1C) with three up-regulated proteins labeled (fold change, Adipoq > E-syt1 > Ivd). To the best of our knowledge, Adipoq has been extensively studied for its role in regulating skeletal muscle metabolism, myofiber type conversion, and skeletal muscle regeneration. In contrast, the effects of E-syt1 on muscle physiology have not been reported or discussed in existing literature.

**Fig. 1:**
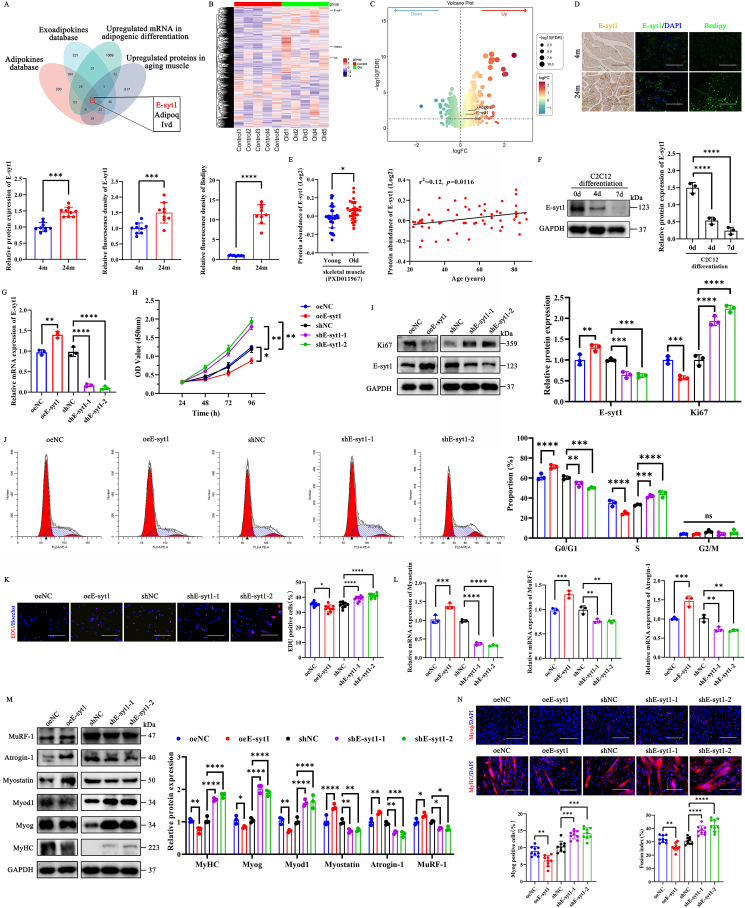
E-syt1 hinders the proliferation and differentiation of myoblasts. (A) Venn diagram illustrating the overlap between adipokines, exoadipokines, mRNA up-regulated during adipogenic differentiation, and protein up-regulated in aging muscle. (B) A heat map was used to reveal three candidate proteins. (C) A volcano plot presents three differentially expressed proteins. (D) Immunohistochemistry, immunofluorescence, Bodipy staining, and quantitative analysis of gastrocnemius muscle samples from young mice (4 months) and old mice (24 months). (E) The E-syt1 expression in young and aged human skeletal muscle (PXD011967). (F) Representative blot images and quantitative analysis of E-syt1 in differentiated differentiated (day 0, 4, 7) C2C12 cells. (G) qRT-PCR validated the efficiency of transfection. (H) CCK8 assay of the oeNC, oeE-syt1, shNC, shE-syt1-1 and shE-syt1-2 groups. (I) Representative blot images and quantitative analysis of Ki67 and E-syt1. (J) FCM for the cell cycle of each group. (K) Representative EDU staining and quantitative analysis of EDU-positive cells. (L) Measurement of mRNA expression of Myostatin, MuRF-1, and Atrogin-1 by qRT–PCR. (M) Representative blot images and quantitative analysis of the myogenic markers (Myod1, Myog, and MyHC) and muscle atrophic factors (MuRF-1, Atrogin-1, and Myostatin). (N) Representative image of immunofluorescence staining and quantitative analysis of Myog and MyHC in each group. (F-J, L-M) n = 3. Values are shown as mean ± SD. (D, K, N) n = 3, three fields per sample were selected. Values are shown as mean ± SD. ∗P < 0.05, ∗∗P < 0.01, ∗∗∗P < 0.001, ∗∗∗∗P < 0.0001. (D, K, N) Scale bar = 200 μm.

Therefore, we first assessed E-syt1 protein expression by immunohistochemistry and immunofluorescence staining, lipid content by Bodipy staining in gastrocnemius muscle (GA) samples from young (4 months) and old (24 months) mice. The results revealed that E-syt1 levels in aged skeletal muscle were significantly greater than those in young muscle, and IMAT was also more pronounced in aged muscle (Fig. 1D and S1A). To verify whether E-syt1 protein is also highly expressed in aging human skeletal muscle, we utilized recent quantitative proteomic sequencing data from individuals of varying ages (PXD011967) [43]. Upon analysis of this dataset, we observed that the abundance of the E-syt1 protein is higher in aged muscle than in young muscle, as well as a mild positive linear correlation between the abundance of protein E-syt1 and age (r^2^ = 0.12, *p* = 0.0116) (Fig. 1E). Afterward, we evaluated the changes in E-syt1 levels during myogenic differentiation, which progressively decreased in C2C12 cells, respectively (Fig. 1F). Although we found that expression of E-syt1 was downregulated during the process of myogenic differentiation, we still needed to determine whether it plays a positive or negative function in myogenic differentiation. Thus, E-syt1 lentiviral vectors were constructed for E-syt1 overexpression (oeE-syt1) or E-syt1 silencing (shE-syt1) in C2C12 cell lines (Fig. S1B) and the overexpression and silencing effects were then evaluated by qRT-PCR (Fig. 1G). On the one hand, overexpression of E-syt1 in C2C12 cells significantly inhibited cell proliferation. In contrast, the silence of E-syt1 accelerated cell proliferation by CCK8 assay (Fig. 1H). Similarly, western blotting and FCM assay showed that the expression of proliferation-related protein Ki67 and the proportion of S-phase cells were significantly decreased in oeE-syt1, while increased dramatically in two different shE-syt1 sequences (Fig. 1I and J). The EDU-positive cell ratio decreased by E-syt1 overexpression and increased by E-syt1 knockdown, as determined by the EDU proliferation assay (Fig. 1K and S1C). On the other hand, the results of qRT‐PCR showed that after overexpression of E-syt1, the relative expression of Myostatin, the E3 ubiquitin ligases MuRF-1 and Atrogin-1 were significantly increased (Fig. 1L), which bind ubiquitin to numerous muscle proteins for proteasomal degradation [44]. Similarly, the WB results showed that E-syt1-mediated muscle atrophy reduces muscle protein synthesis and increases muscle protein degradation via the ubiquitin-proteasome pathway. This effect is attenuated by E-syt1 expression downregulation (Fig. 1M). For myogenic differentiation, immunofluorescence staining for Myog and MyHC (Fig. 1N–S1D and S1E) demonstrated that silencing E-syt1 led to a notable enhancement in the expression of Myog, as well as the myotube formation. Conversely, overexpressing E-syt1 had the opposite effect.

### 3.2 E-syt1 suppresses both mitochondrial respiration and biogenesis in C2C12 myoblasts

The above data indicated that E-syt1 is downregulated during the differentiation of C2C12 cells. Recent work demonstrated that E-syt1 recruited in the EMCS regulates mitochondrial membrane biogenesis, mitochondrial lipid transport, and respiration [11,45]. Kubat et al. and Kadoguchi et al. have shown that mitochondrial dysfunction and skeletal muscle atrophy are closely related [46,47]. In the study, we investigated further the effects of E-syt1 on the respiration and biogenesis of mitochondria. First, we examined the expression level of several subunits of the mitochondrial respiratory chain complexes (NDUFS1 of complex I; SDHA of complex II; UQCRC2 of complex III; COXIV of complex IV; ATP5A1 of complex V) and mitochondrial biogenesis using western blotting (Fig. 2A). E-syt1 overexpression downregulates the levels of complex I, II, III, and IV. The paroxysm proliferate-activated receptor-gamma activator 1 (PGC-1) family, including PGC-1α and PGC-1β, are critical transcriptional coactivators regulating mitochondrial biogenesis in mammals [[48], [49], [50]]. In contrast, E-syt1 knockdown increased the levels of mitochondrial respiratory chain complexes and PGC-1α and PGC-1β expression (Fig. 2A and B). Subsequently, we employed OCR to assess the mitochondrial respiration capacity in C2C12 cells. As shown in Fig. 2C–E, when OCR was normalized to CS activity, overexpression of E-syt1 resulted in a further reduction in both maximal and spare respiratory capacity, and the ablation of E-syt1 significantly increased the OCR in C2C12 cells. Nevertheless, no significant variations were observed in the baseline, ATP-linked, proton leak, or non-mitochondrial respiration. Meanwhile, the study followed a notable decrease in the ratio of mtDNA to nuclear DNA (nDNA) in the oeE-syt1 group. In contrast, the shE-syt1 group showed an increase in this ratio compared to the control group (Fig. 2F). Furthermore, the mitochondrial membrane potential (MMP) is pivotal in maintaining mitochondrial homeostasis by facilitating the transportation of essential proteins and ions required for proper mitochondrial functioning. Alterations in the mitochondrial membrane potential can indicate mitochondrial dysfunction [23]. Using TMRE staining, which is sequestered by active mitochondria in live cells but leaks out of depolarised mitochondria, we examined whether E-syt1 affects mitochondrial depolarization. The results showed that oeE-syt1 treatment induced a decreased MMP, whereas shE-syt1 treatment resulted in an increased MMP (Fig. 2G).

**Fig. 2:**
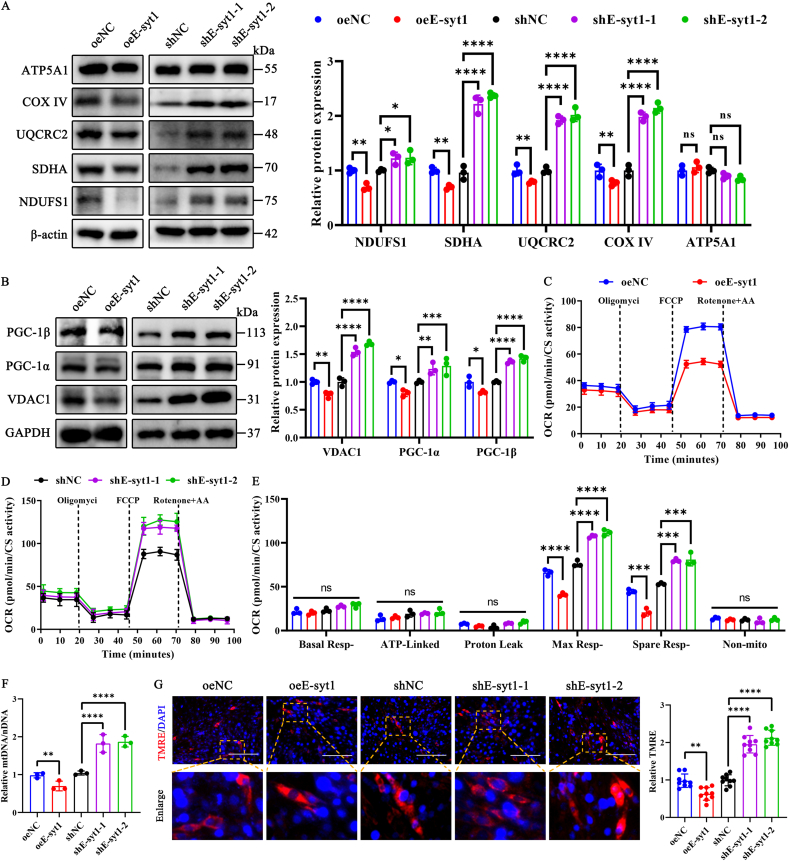
E-syt1 suppresses both mitochondrial respiration and biogenesis in C2C12 myoblasts. (A) Representative blot images and quantitative analysis of OXPHOS complexes (NDUFS1, SDHA, UQCRC2, COX IV, and ATP5A1). (B) Representative blot images and quantitative analysis of mitochondrial biogenesis markers (VDAC1, PGC-1α, and PGC-1β). (C) Representative oxygen consumption curves in the oeNC and oeE-syt1-treated C2C12 cells. (D) Representative oxygen consumption curves in the shNC and shE-syt1-treated C2C12 cells. (E) Quantification analysis of basal respiration, ATP-linked respiration, proton leak respiration, maximal respiration, spare respiration, and non-mitochondrial respiration in overexpression or silencing cells. (F) Genomic DNA was extracted from the overexpression or silencing of C2C12 myoblasts separately. The ratio of mitochondrial DNA and nuclear DNA determined mitochondrial content. (G) Representative images and quantitative analysis of the mitochondrial membrane potential. The bottom row is a magnified view of the area in the image from the top row, indicated with a yellow square. (A–F) n = 3. Values are shown as mean ± SD. (G) n = 3, three fields per sample were selected. Values are shown as mean ± SD. Ns, no significance, ∗P < 0.05, ∗∗P < 0.01, ∗∗∗P < 0.001, ∗∗∗∗P < 0.0001. (G) Scale bar = 200 μm.

### 3.3 E-syt1 overexpression impaired mitochondrial dynamics in myoblasts

Mitochondrial dynamics are defined by the frequent fusion and division of mitochondria within cells [51]. To investigate whether E-syt1 directly alters C2C12 myoblast's mitochondrial dynamics, we isolated mitochondria from transfected cells and assessed the protein expression of components of mitochondrial dynamics. Concerning mediators of mitochondrial fusion, E-syt1 overexpression decreased the expression of the two outer mitochondrial membrane proteins, mitofusin 1 (Mfn1) and mitofusin 2 (Mfn2), an inner mitochondrial membrane protein, dynamin-like GTPase (Opa1), but increased them in shE-syt1-treated C2C12 cells. As expected, the fission proteins dynamin-related protein 1 (Drp1) and fission 1 protein (Fis1) exhibited enhanced expression in oeE-syt1-infected C2C12 cells but were reduced in expression in shE-syt1-treated cells (Fig. 3A). Mitochondria are dynamic organelles that undergo continuous fusion and fission; the balance between fission and fusion dictates the overall morphology of the mitochondria [52]. Hence, we utilized MTR staining to examine mitochondrial structure in each group of C2C12 cells. As illustrated in Fig. 3B, overexpression of E-syt1 resulted in transforming the mitochondrial shape from a network of filaments to a more punctate and fragmented structure, together with a reduction in fluorescence emitted by MTR. Nevertheless, cells treated with shE-syt1 improved the abnormal mitochondrial characteristics and enhanced the density of MTR fluorescence. By examining the mitochondrial morphologic parameters in different groups, we discovered that cells treated with E-syt1 overexpression exhibited poorer mitochondrial structure compared to the control group. Conversely, when E-syt1 was silenced, the abnormal mitochondrial structure was reversed (Fig. 3B). Simultaneously, we evaluated the overall connectivity and structural complexity. We quantified these aspects using metrics such as branch count, branch junction count, branch length, and average branch length in the skeletonized mitochondrial network by the Mitochondria Analyzer plugin in ImageJ (NIH). The mitochondrial network characteristics of cells treated with oeE-syt1 were decreased compared to the control group. Still, the parameters of cells treated with shE-syt1 were improved compared to the control group (Fig. 3C). E-syt1 has long been regarded as a critical regulator of intracellular calcium homeostasis. Recent findings indicate that E-syt1 is also crucial for Ca^2+^ transport at EMCS. It was reported that mitochondrial calcium overload caused by calcium influx leads to the loss of MMP and oxidative damage [53]. Rhod-2 AM and MitoSOX are effective fluorescence probes widely used to determine the mitochondrial calcium ions and mitochondrial-specific ROS, respectively [53]. Our results showed that mitochondrial Ca^2+^ and ROS significantly increased after E-syt1 overexpression, while mitochondrial calcium content was slightly reduced when E-syt1 was silenced (Fig. 3D and S2A). These results demonstrate that E-syt1 overexpression leads to mitochondrial calcium overload, mitochondrial ROS burst, and ultimately, mitochondrial dysfunctions in C2C12 cells. Afterward, we performed TEM to assess the influence of E-syt1 on changes in mitochondrial morphology. TEM images revealed that the number of damaged mitochondria increased significantly, accompanied by an increase in the number of autophagosomes in the oeE-syt1 group. These autophagosomes were observed to engulf mitochondria, facilitating mitophagosome production. Conversely, shE-syt1-treated cells showed a significantly higher number of morphologically normal mitochondria than shNC (Fig. 3E).

**Fig. 3:**
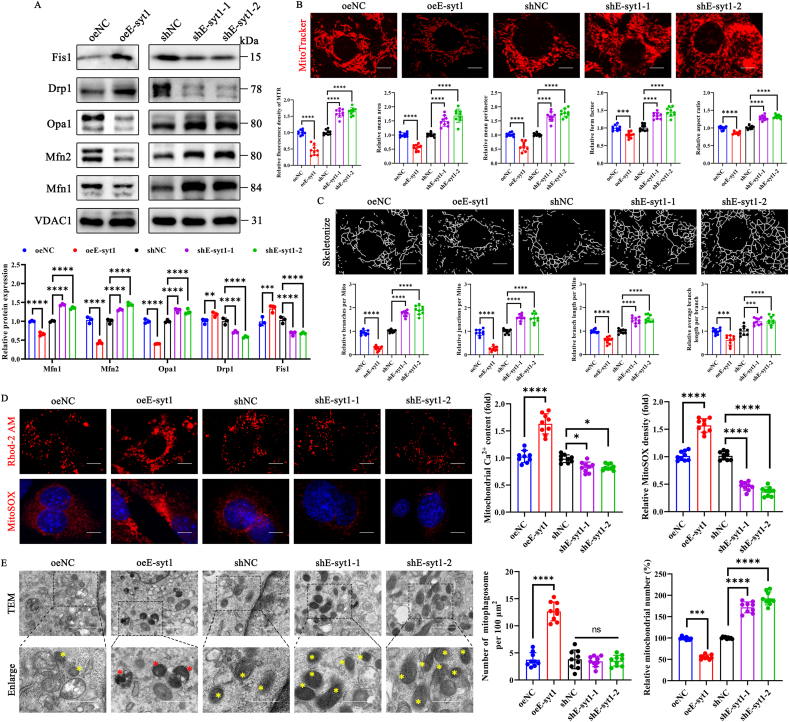
E-syt1 impaired mitochondrial dynamics in myoblasts. (A) Representative blot images and quantitative analysis of mitochondrial fusion proteins (Mfn1, Mfn2, Opa1) and fission proteins (Drp1, Fis1). (B) Representative images of mitochondria labeled with Mito-Tracker Red (100 nM, 30 min)., and 2D morphological analysis of each group. (C) Skeletonization of the mitochondrial objects identified in B and quantitative analysis of mitochondrial network connectivity. (D) Representative images of Rhod-2 AM-stained mitochondrial Ca^2+^ and MitoSOX-stained mitochondrial ROS. Quantitative analysis of the content of mitochondrial Ca^2+^ and relative ROS production. (E) Representative images of transmission electron microscopy. The bottom row is a magnified view of the area in the image from the top row, indicated with a black square (Yellow asterisks represented normal mitochondria. Red asterisks represented mitophagosome). Quantitative analysis of the number of mitophagosomes and morphologically normal mitochondria. (A) n = 3. Values are shown as mean ± SD. (B–E) n = 3, three fields per sample were selected. Values are shown as mean ± SD. Ns, no significance, ∗∗P < 0.01, ∗∗∗P < 0.001, ∗∗∗∗P < 0.0001. (B–D) Scale bar = 10 μm. (E) Top scale bar = 1 μm, bottom scale bar = 500 nm.

### 3.4 E-syt1 overexpression inhibits mitophagic flux

**Fig. 4:**
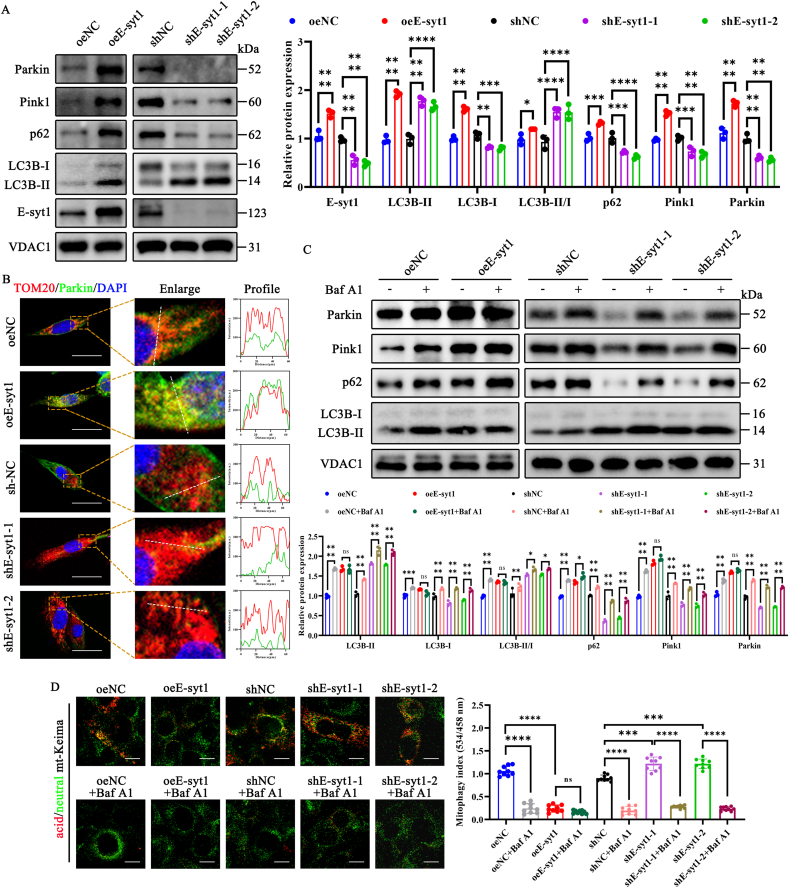
Overexpression of E-syt1 suppressed mitophagic flux. (A) Representative blot images and quantitative analysis of E-syt1, LC3B–I, LC3B-II, p62, Pink1, and Parkin. (B) Representative images of mitochondria labeled with red (TOM20) and mitophagy labeled with green (Parkin) were utilized. Profiles were obtained using ImageJ software along the white dashed line. (C) Representative blot images and quantitative analysis of LC3B–I, LC3B-II, p62, Pink1, and Parkin in groups with or without treatment with Baf A1. (D) Representative mt-keima image and quantitative analysis of mitophagy index (534/458 nm) in each group. (A, C) n = 3. Values are shown as mean ± SD. (B, D) n = 3, three fields per sample were selected. Values are shown as mean ± SD. Ns, no significance, ∗P < 0.05, ∗∗P < 0.01, ∗∗∗P < 0.001, ∗∗∗∗P < 0.0001. (B) Scale bar = 20 μm. (D) Scale bar = 10 μm.

Mitophagy is a type of macro-autophagy that promotes mitochondrial renewal and maintains mitochondrial homeostasis by selectively removing dysfunctional mitochondria [16,17,20]. Hence, we next investigated the impact of E-syt1 overexpression or silencing on mitophagy in myoblasts. First, we found that E-syt1 expression in myoblast mitochondrial extracts has an effective overexpression and silencing effect following cell transfection (Fig. 4A). Following treatment with oeE-syt1, the levels of the LC3B II/I ratio, Parkin, and Pink1 all exhibited an increase. However, the level of p62 expression was notably increased, suggesting a probable inhibition of mitophagic flux. Conversely, the levels of Parkin, Pink1, and p62 were reduced, and the LC3B II/I ratio remained elevated in shE-syt1-treated cells (Fig. 4A). When the MMP was damaged, the pathway for PINK1 to enter the mitochondrial inner membrane was blocked, causing PINK1 to stably accumulate in the mitochondrial outer membrane. PINK1 recruits Parkin to the mitochondria, after which Parkin catalyzes the ubiquitination of mitochondrial proteins, triggering Parkin-mediated mitophagy [54]. As expected, confocal imaging and fluorescence intensity profiles demonstrated an apparent co-localization between Parkin and TOM20 in oeE-syt1-treated cells (Fig. 4B and S2B). Based on the accumulation of mitophagosomes revealed by TEM images, we examined whether E-syt1 inhibits mitophagic flux using autophagy flux inhibitor bafilomycin A1 (Baf A1). The levels of the LC3B-II were increased in cells treated with Baf A1 due to the prevention of lysosomal degradation. However, Baf A1 treatment did not further enhance the LC3B-II/I ratio, Pink1, and Parkin protein levels in oeE-syt1 cells, although p62 expression was slightly increased. Furthermore, we also found that p62, LC3B-II/I ratio, Pink1, and Parkin protein levels were increased in cells treated with Baf A1 and sh-Esyt1 (Fig. 4C). These results indicate increased LC3B-II under baseline conditions (without Baf A1 treatment) because E-syt1 overexpression blocks the mitophagic flux. To provide additional evidence for the effects of E-syt1 on mitophagic flux, we monitored mt-Keima. High levels of acidic mitochondria were found in cells treated with oeNC. Nevertheless, applying oeE-syt1 to the cells decreased the quantity of acidic mitochondria. In contrast, the shE-syt1 group exhibited an increased mitophagy index compared to the shNC group, suggesting that mitophagy activity was enhanced in the shE-syt1 group (Fig. 4D and S2C). The mitophagy index of cells in all groups, except for the oeEsyt1 group, decreased considerably following the administration of Baf A1. These data indicate that E-syt1 overexpression inhibits mitophagic flux, impairing mitochondrial degradation.

### 3.5 E-syt1 inhibits the fusion of mitophagosomes with lysosomes and lysosomal activity

**Fig. 5:**
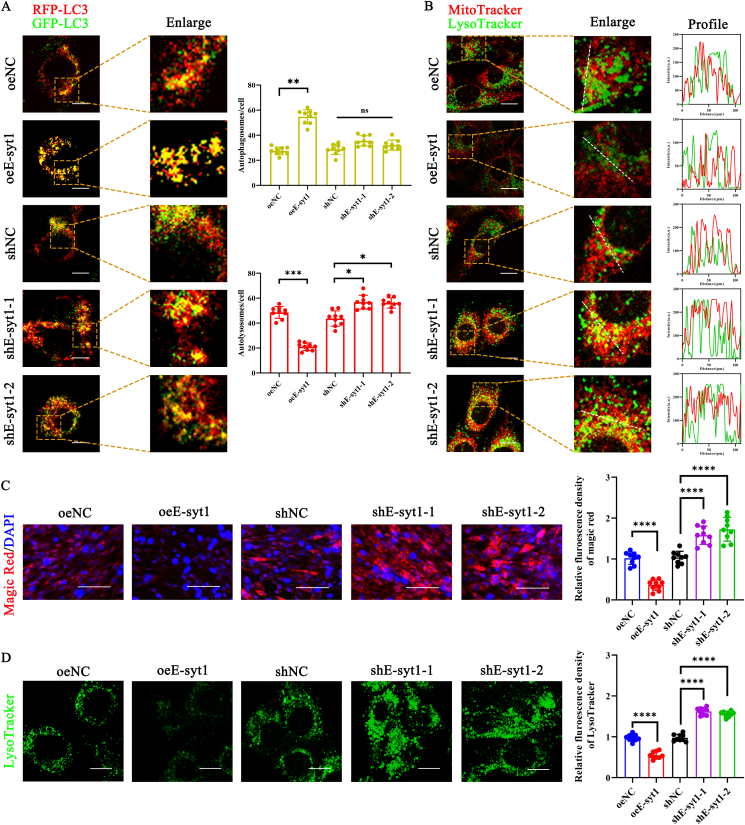
E-syt1 inhibits the fusion of mitophagosomes with lysosomes and lysosomal activity. (A) The cells were transfected with GFP-LC3-RFP. The enlarged images are magnified from the boxed areas in the overlay images. Quantification of autophagosomes (yellow dots) and autolysosomes (red dots) in cells transfected with GFP-LC3-RFP was analyzed. (B) Representative co-localization images of MitoTracker and LysoTracker in each group. Profiles were obtained using ImageJ software along the white dashed line. (C) Magic Red, representative images and quantitative analysis of lysosomal cathepsin B activity analyzed the lysosomal activity and acidity. (D) Representative images and quantitative analysis of lysosome acidity. (A–D) n = 3, three fields per sample were selected. Values are shown as mean ± SD. ∗∗∗∗P < 0.0001. (A, B, D) Scale bar = 10 μm. (C) Scale bar = 200 μm.

Autophagic flux may be impeded by a reduction in lysosomal hydrolase activity resulting from elevated lysosomal pH or by the inhibition of autophagosome-lysosome fusion [55]. oeE-syt1/shE-syt1 and oeNC/shNC cells were then transfected with GFP-LC3-RFP, an autophagic flux reporter in which GFP-LC3, but not RFP-LC3, is sensitive to degradation in the acidic environment of the autolysosome [55]. Yellow puncta (positive for both GFP and RFP signals) represent autophagosomes or un-acidified amphisomes, whereas red-only puncta are acidified amphisomes or autolysosomes. We observed that more yellow dots were accumulated in oeE-syt1 cells compared with oeNC cells (Fig. 5A and S2D). As depicted in Fig. 3E, cellular electron microscopy findings indicated a considerable increase in the number of mitophagosomes, but not autolysosomes, in oeE-syt1-treated cells. This phenomenon could result from impaired fusion of autophagosomes and lysosomes or increased lysosomal pH. To examine the fusion events between autophagosomes and lysosomes, we analyzed the co-localization of MitoTracker and LysoTracker. Our results revealed a notable reduction in the co-localization of mitochondria and lysosomes in cells treated with oeE-syt1. Nonetheless, the fluorescence intensity profile indicated enhanced co-localization after silencing E-syt1 (Fig. 5B and S2E). To investigate lysosomal activity and assess the acidic environment of lysosomes, we employed Cathepsin Magic Red and LysoTracker, which are cleaved by active cathepsins to yield a fluorescent product and a pH-sensitive dye that exhibits increased fluorescence in acidic organelles, respectively. E-syt1 overexpression substantially reduced the fluorescence intensity, supporting the result of the lowered cathepsin B activity (Fig. 5C and S2F). Fig. 5D shows a significant increase in lysosomal pH in cells treated with oeE-syt1, but a decrease was observed in sh-esyt1-treated cells. These data suggest that E-syt1 blocks the binding of mitophagosomes to lysosomes and impedes the acidification of lysosomes, thereby inhibiting lysosomal degradation.

### 3.6 Silencing of E-syt1 increased the capacity of endurance exercise and muscle mass in OVX mice (estrogen deficiency-induced muscle atrophy)

**Fig. 6:**
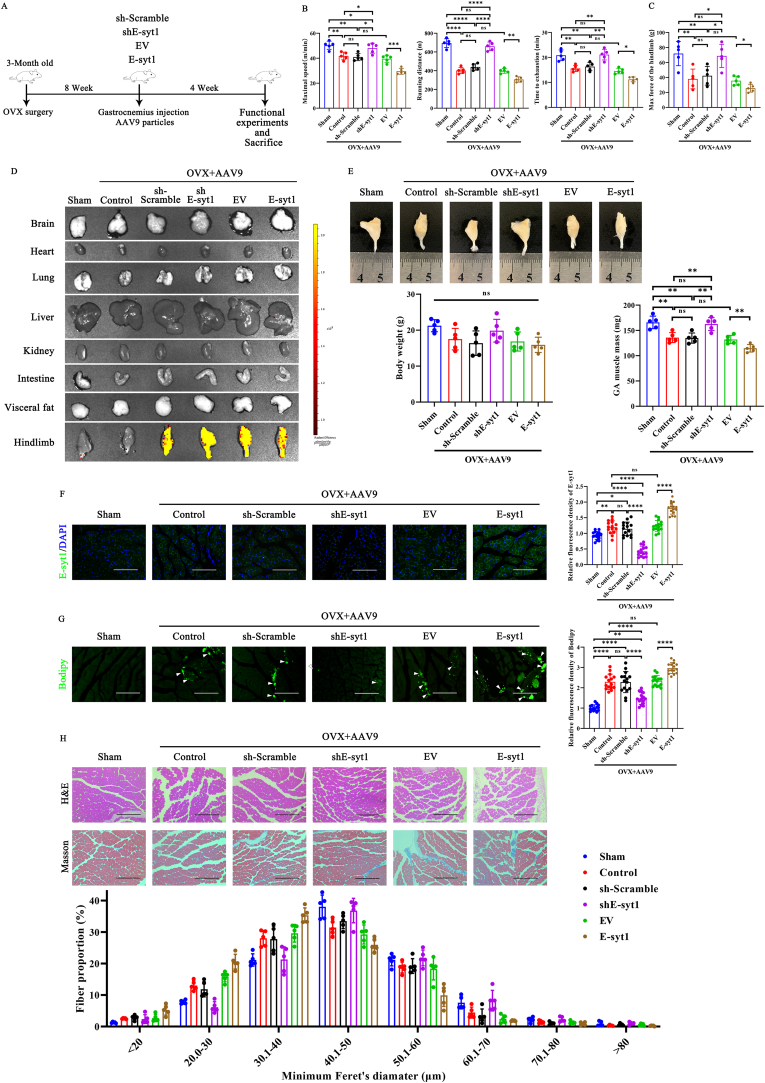
Lack of E-syt1 improved physical performance and muscle quality in OVX mice. (A) Schematic diagram of animal experiments. (B) Physical performance assessment on a treadmill. After four weeks of treatment, measurements of maximal running speed, distance, and running time. (C) Grip strength was measured after treatment. (D) EGFP expression in individual tissues was monitored by IVIS-100 optical imaging 4 weeks post-injection. (E) Representative macro photographs of GA and body weight and GA mass. (F) Representative image of immunofluorescence staining and quantitative analysis of E-syt1 in each group. (G) Representative image of Bodipy staining and quantitative analysis of the fluorescence intensity of Bodipy. (H) A representative image of H&E and Masson staining and quantitative analysis of the minimum ferret diameter of GA myofibers. (B–C, E, H) n = 5. Values are shown as mean ± SD. (F, G) n = 5, three fields per sample were selected. Values are shown as mean ± SD. Ns, no significance, ∗P < 0.05, ∗∗P < 0.01, ∗∗∗P < 0.001, ∗∗∗∗P < 0.0001. (F, G) Scale bar = 200 μm.

Previous studies have shown that OVX surgery can result in muscle atrophy, an increase in skeletal muscle intramyocellular lipids, and a higher percentage of body fat compared to sham-operated animals [[56], [57], [58]]. OVX rodents are an excellent experimental model for SO because they exhibit an obesity phenotype and sarcopenia [59,60]. Thus, to further verify the effects of E-syt1 in vivo, we entailed AAV9-mediated intramuscular (i.m.) delivery of sh-Scramble, shE-syt1, EV, and E-syt1 on the OVX mice (Fig. 6A). We estimated the capacity for physical exercise in Sham, Control, AAV9-sh-Scramble, AAV9-shE-syt1, AAV9-EV, and AAV9-E-syt1 mice using a treadmill exhaustion test (Fig. 6B). As expected, the maximal running speed, running distance, and time to running exhaustion of control mice were substantially lower than those of sham mice, and the performance of AAV9-sh-Scramble and AAV9-EV mice was comparable to that of control mice. Interestingly, the capacity for physical exercise was improved in OVX mice with AAV9-shE-syt1 treatment compared to that in OVX mice treated with PBS or AAV9-sh-Scramble. However, AAV9-E-syt1 injection further decreased the capacity for physical exercise. The same changes were seen in the max force of the hindlimb (Fig. 6C). In comparison to the sham and control groups, IVIS imaging revealed that the AAV9-delivered viral particles exhibited no significant off-target effects, predominantly accumulating around the hindlimb, with no viral expression detected in the brain, heart, lungs, liver, kidneys, intestines, or visceral fat (Fig. 6D**)**. Meanwhile, AAV9-shE-syt1 treatment increased the mass of the gastrocnemius compared to the AAV9-sh-Scramble group as determined by measurements of macrophotographs, body mass, and gastrocnemius mass. The administration of AAV9-E-syt1 resulted in an additional decrease in both muscle mass in OVX mice (Fig. 6E). Animals were sacrificed for slice preparation and histology assessment. First, we confirmed the effect of silencing or overexpressing E-syt1 in vivo by immunofluorescence staining (Fig. 6F and S3A). Then, we observed elevated E-syt1 expression and intramyocellular lipids in mice following OVX surgery, which were significantly reduced by AAV9-shE-syt1 injection. However, when E-syt1 was overexpressed, intermuscular fat infiltration was further aggravated (Fig. 6G). The minimum ferret diameter of myofibers of GA increased in the AAV9-shE-syt1 group and decreased in the AAV9-E-syt1 group, compared to the AAV9-shScramble and AAV9-EV groups, respectively (Fig. 6H**)**.

### 3.7 E-syt1 ablation improved mitochondrial homeostasis and increased the proportion of oxidative muscle fibers

**Fig. 7:**
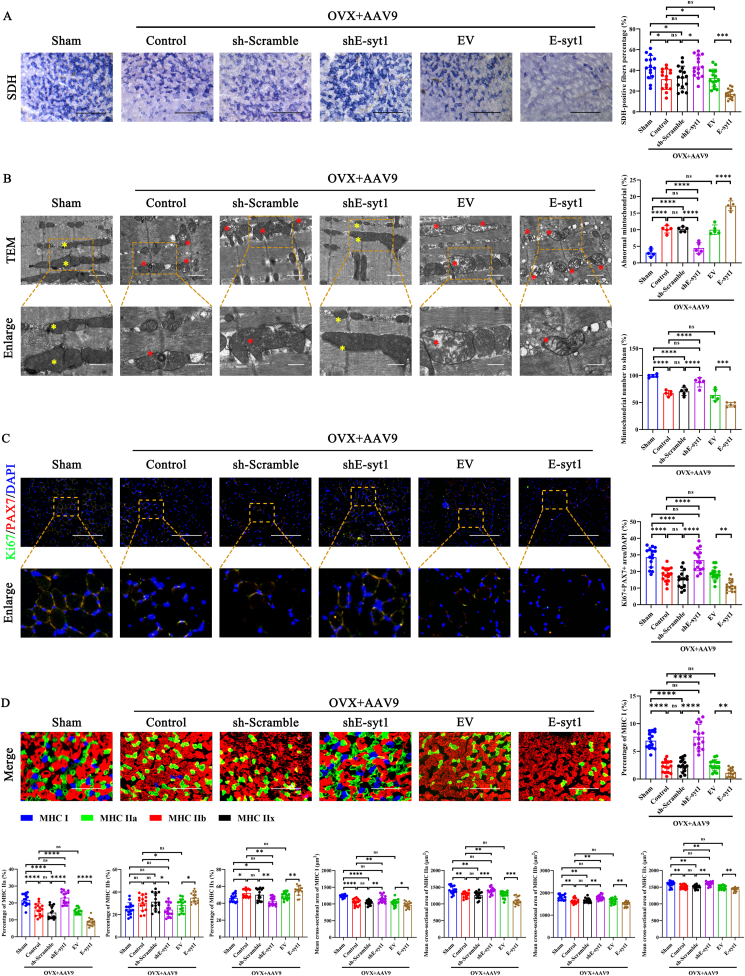
E-syt1 ablation improved mitochondrial homeostasis and skeletal muscle remodeling in OVX mice. (A) Representative SDH staining and quantitative analysis of SDH-positive fibers percentage of each group. (B) Electron microscopy examination was performed on the GA samples. The bottom row, a magnified view of the area in the image from the top row, is indicated with a yellow square. Yellow asterisks represented normal mitochondria. Red asterisks represented abnormal mitochondria. Quantitative analysis of the abnormal mitochondria and mitochondrial number ratio in each group. (C) Representative image of immunofluorescence staining and quantitative analysis of PAX7 and Ki67. (D) Representative image of immunofluorescence staining and quantitative analysis of MHC I, IIa, IIb, and IIx myofibers. (B) n = 5. Values are shown as mean ± SD. (A, C, D) n = 5, three fields per sample were selected. Values are shown as mean ± SD. Ns, no significance, ∗P < 0.05, ∗∗P < 0.01, ∗∗∗P < 0.001, ∗∗∗∗P < 0.0001. (A, C, D) Scale bar = 200 μm. (B) Top scale bar = 1 μm, bottom scale bar = 500 nm.

It is widely recognized that skeletal muscle fibers have various muscle fiber types. Fast fibers depend more on glycolysis for energy production, while slow fibers tend to have more mitochondria and a metabolic pattern mainly controlled by oxidative phosphorylation [61]. To assess the experimental and control groups' metabolic condition, we analyzed the muscle fibers' characteristics related to oxidation and glycolysis using SDH staining. As depicted in Fig. 7A, compared to the sham group, the control, AAV9-shScramble, and AAV9-EV groups exhibited reduced SDH activity, which was recovered by AAV9-shE-syt1 treatment and considerably suppressed by AAV9-E-syt1 treatment. TEM analysis of muscles revealed an increase in the proportion of abnormal mitochondria, including mitochondria with disrupted membranes, loss of cristae, and vacuolization. TEM analysis further verified that the morphology and number of mitochondria were partially improved after AAV9-shE-syt1 treatment. Still, the administration of E-syt1 only exacerbated mitochondrial damage and dysfunction (Fig. 7B). In addition, immunofluorescence co-staining of PAX7 and Ki67 showed that E-syt1 ablation promoted the proliferation of muscle satellite cells in OVX mice. In contrast, AAV9-E-syt1 administration had the opposite effect (Fig. 7C and S3B). These findings indicate that E-syt1 ablation contributes to mitochondrial network remodeling and muscle stem cell pool maintenance. Furthermore, immunofluorescence staining revealed a reduction in the proportion of oxidative fibers (MHC I and MHC IIa) in the muscles of both the control and AAV9-shScramble groups, which was alleviated following the administration of AAV9-shE-syt1. Conversely, the injection of AAV9-E-syt1 exacerbated the loss of oxidative fibers and was accompanied by increased glycolytic fibers (MHC IIb and MHC IIx). On the other hand, our findings indicate that AAV9-shE-syt1 treatment significantly enhanced the mean cross-sectional area of various myofiber types, whereas AAV9-E-syt1 treatment resulted in a reduction in the mean cross-sectional area of myofiber in OVX mice with varying degrees (Fig. 7D and S3C). These results implied that the lack of E-syt1 protected against muscle atrophy by increasing the oxidative capacity of muscle fibers, maintaining mitochondrial homeostasis, increasing the distribution of oxidative fibers, and remodeling skeletal muscle fiber in OVX mice.

## 4 Discussion

**Fig. 8:**
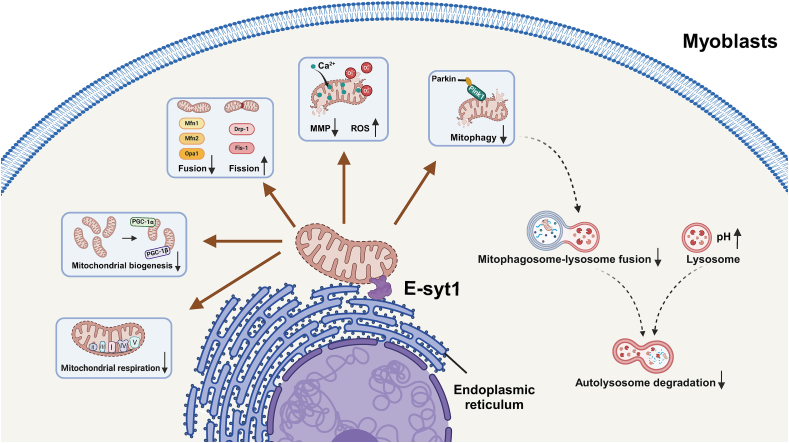
Schematic figure showing that the E-syt1 is a negative regulator of muscle function. E-syt1 hinders myoblast mitochondrial respiration and biosynthesis, reduces mitochondrial membrane potential, promotes mitochondrial fission, and results in mitochondria calcium overload and mitochondrial ROS burst. Meanwhile, E-syt1 also inhibits mitophagosome fusion with lysosomes and lysosomal acidification, reducing mitophagic flux.

The current work proved that E-syt1 played a crucial role in SO. Mechanistically, E-syt1 overexpression inhibits mitochondrial respiration and biogenesis, promotes mitochondrial fission, and results in mitochondrial calcium overload, mitochondrial ROS burst and ultimately induces mitochondrial dysfunctions. Meanwhile, E-syt1 hinders mitophagosome binding to lysosomes, obstructs the degradation of autophagic substrates, and reduces mitophagic flux (Fig. 8). OVX animals demonstrate an increase in body weight and a reduction in lean body mass and energy expenditure. Consequently, despite not precisely replicating the sarcopenia associated with aging, they serve as a viable preclinical model for studying SO [62]. Suppression of E-syt1 in OVX mice (estrogen deficiency-induced muscle atrophy) leads to a substantial restoration of gastrocnemius mitochondrial function, muscle mass, and oxidative capacity of the muscle fibers. Our data suggests that E-syt1 deficiency is essential for enhanced mitochondrial function and skeletal muscle remodeling in SO treatment.

Myogenesis includes myoblast proliferation, differentiation, and myotube formation [[63], [64], [65]]. Cai et al. reported that the inhibition of MLL1 resulted in myoblast proliferation suppression and myoblast differentiation impairment due to reduced levels of Myf5 and Cyclin D1 expression [63]. Multiple studies have demonstrated that satellite cells' proliferative and differentiative capacities in mature myofibers decline with age [66]. This study found that E-syt1 expression is downregulated with myogenic differentiation, indicating that E-syt1 may be involved in myoblast differentiation. To elucidate the mechanism by which E-syt1 regulates skeletal muscle, we first determined excessive expression of E-syt1 in C2C12 myoblasts hinders myoblast proliferation and myotube formation, whereas suppression of E-syt1 enhances these processes. Skeletal muscle is the most metabolically active and highly structured tissue, and mitochondrial dysfunction is already a well-known feature closely associated with the loss of muscle function, thereby making maintaining a functional mitochondrial system critical in sarcopenia [67]. Janer et al. provided that E-syt1 plays a role in tethering endoplasmic reticulum and mitochondria and induces a significant alteration of the mitochondrial lipidome [68]. According to Pan et al., the mitochondria of ANKRD22-expressing 293T cells exhibited swelling, vacuolar-like alterations, and diminished mitochondrial cristae after E-syt1 overexpression [12]. Then, we monitored the skeletal muscle's mitochondrial function and energy metabolism with overexpression or silencing of E-syt1 expression. Interestingly, our data showed that E-syt1 overexpression downregulates the levels of mitochondrial complex I-IV and mitochondrial biogenesis proteins PGC-1α and PGC-1β, whereas the silencing of E-syt1 improves them. PGC-1α and PGC-1β are widely recognized as critical regulators of mitochondrial metabolism and biogenesis [69]. They activate transcription factors (NRF-2 and TFAM) encoded by nuclear DNA and mtDNA, leading to mitochondrial biogenesis [70]. Similarly, the increase in the mtDNA/nDNA ratio after shE-syt1 treatment also confirms the increase in mitochondrial content. Due to the potential impact of increased mitochondrial content on the assessment of cellular respiration state, we employed citrate synthase activity to standardize the OCR results. The findings verified that suppressing E-syt1 enhances mitochondrial respiration in myoblasts.

Some studies have found that altering the mitochondrial network potentially contributes to the decline in muscle mass [17,25,71]. It is imperative to meticulously synchronize the fusion and fission processes of mitochondria and regulate the turnover of mitochondria to uphold a robust mitochondrial population and avert the emergence of pathological states [18]. Our western blotting analysis confirmed that mitochondrial dynamics as indicated by increased mitochondrial fission (Drp1 and Fis1) and reduced mitochondrial fusion (Mfn1, Mfn2, and Opa1) processes in C2C12 cells using overexpressed E-syt1. Mitochondrial fission plays a role in maintaining cellular quality control by dividing the interconnected mitochondrial network and separating components that have lost their membrane potential [18]. Similarly, more fragmented mitochondria were found in the oeE-syt1 myoblasts according to confocal imaging. Analysis of mitochondrial network parameters also confirmed that E-syt1 overexpression would damage mitochondria. Calcium overload is closely related to mitochondrial dysfunction and mitochondrial membrane permeability. This paper found that mitochondrial calcium overload and ROS release were responsible for mitochondrial homeostasis disruption. Our analysis of the mitochondrial electron microscopy results also revealed considerable damage to the mitochondria in the oe-Esyt1 group. When inspected under electron microscopy, it was found that autophagosomes surrounded most of the mitochondria, and mitophagosomes accumulated. However, an equivalent number of lysosomes was not seen, given that degradation blockade contributed to autophagosome accumulation.

Mitophagy is a form of macro-autophagy that involves the targeted elimination of dysfunctional and depolarised mitochondria from healthy skeletal muscle cells through the autophagy-lysosome complex [16]. In this study, the cells were treated with Baf A1. We further identified that oeE-syt1 suppressed mitophagic flux in vitro. Liu et al. proposed that mt-Keima is more sensitive than mito-QC for Pink1-Parkin mitophagy both in cell culture and in vivo [35]. Therefore, we further confirmed that overexpression of E-syt1 led to decreased mitophagy using the mt-keima fluorescent probe. Evidence indicates that ROS regulates autophagy variably based on the physiological context. Oxidative stress resulting from mitochondrial or lysosomal malfunction hinders autophagy, as demonstrated in cellular models of aging and senescence [55]. We proposed that mitochondrial ROS may be accountable for the impairment of lysosomal acidity. We evaluated lysosomal cathepsin B activity using the Magic Red reagent and lysosomal acidity with the pH-sensitive dye LysoTracker. The data indicated that E-syt1 overexpression elevated lysosomal pH and compromised lysosomal functionality. Moreover, tandem GFP-LC3-RFP experiments robustly indicated that E-syt1 overexpression impeded autophagosomal-lysosomal fusion. Collectively, these findings suggest that E-syt1 diminishes mitophagic flux in myoblasts.

TEM images found more abnormal mitochondria in the gastrocnemius from OVX-Control mice than Sham mice. Additionally, following one month of AAV9-shE-syt1 treatment, mitochondrial morphology was restored to a compact, regular mitochondrial morphology resembling that seen in the Sham group. However, the acquisition of E-syt1 further exacerbates mitochondrial dysfunction. Evidence indicates a reduced proportion of oxidative muscle fibers and increased glycolytic fibers correlate with a higher body mass index [72]. Similarly, we observed a decrease in the proportion of oxidative muscle fibers and an increase in IMAT in OVX mice. Skeletal muscle fibers experience significant structural and metabolic alterations in response to normal or pathological stimuli. In skeletal muscle, upregulation of mitochondrial biogenesis may be associated with altering muscle fiber type toward the more oxidative type I and IIa fibers [61,[73], [74], [75], [76]]. The mitochondrial system is tightly coupled to the muscle contractile fiber type, and its metabolic type plays a crucial role in the muscle fiber type transformation process. Our histochemical staining for SDH, MHC I, and MHC IIa, which are hallmarks of oxidative metabolism, revealed that the loss of function of E-syt1 reverses muscle fiber type transition in OVX mice with estrogen deficiency-induced muscle atrophy. The shE-syt1 therapy augmented the cross-sectional area of various muscle fiber types, maybe because of E-syt1 on overall mitochondrial activity. The collective findings of the in vivo studies prove that the deficiency of E-syt1 contributes to attenuating SO by improving mitochondrial morphology, oxidative properties, increasing fiber size, and remodeling skeletal muscle fiber.

## 5 Limitations

Our in vitro and in vivo findings demonstrate that E-syt1 overexpression impedes mitophagic flux, with this reduction linked to lysosomal malfunction. However, the exact mechanism of E-syt1-induced defective autophagy remains obscure. Furthermore, our study did not ascertain the primary origin of the E-syt1 protein, nor did it determine whether E-syt1 facilitates crosstalk between adipose tissue and skeletal muscle. Therefore, our future research will focus on conducting detailed mechanistic investigations. The animal model used in this study is an estrogen deficiency-induced SO model, and there is a lack of discussion on the possible effects of gender differences on the results. Furthermore, disparities persist in the choice of animal models for OVX mice and SO induced by a high-fat diet. In the future, we will further verify our conclusions in multiple animal models. Finally, we aspire to conduct further investigations utilizing muscle-specific knockout mice, as this would enhance the persuasiveness of our conclusions.

## 6 Conclusion

In summary, E-syt1 functions as a negative regulator of myogenesis. The overexpression of E-syt1 impedes myoblast proliferation, differentiation, mitochondrial respiration, and biosynthesis. Simultaneously, it induced calcium overload, elevated mitochondrial ROS, caused lysosomal dysfunction, and disrupted autophagic flux. In vivo tests revealed that the suppression of E-syt1 markedly enhanced myofibers' endurance exercise ability, muscle mass, mitochondrial function, and oxidative capacity in OVX mice. This study establishes that E-syt1 is a novel contributor to the etiology of skeletal muscle metabolic abnormalities in SO, and addressing E-syt1-induced dysfunction may represent a viable way to mitigate SO.

## CRediT authorship contribution statement

**Chao Song:** Writing – review & editing, Writing – original draft, Visualization, Software, Project administration, Data curation. **Wu Zheng:** Software, Methodology. **Guoming Liu:** Software, Methodology. **Yiyang Xu:** Software, Data curation. **Zhibo Deng:** Software, Data curation. **Yu Xiu:** Methodology. **Rongsheng Zhang:** Software, Formal analysis. **Linhai Yang:** Software, Formal analysis. **Yifei Zhang:** Validation, Software. **Guoyu Yu:** Validation, Formal analysis. **Yibin Su:** Methodology. **Jun Luo:** Resources. **Bingwei He:** Writing – review & editing, Supervision, Funding acquisition, Conceptualization. **Jie Xu:** Writing – review & editing, Supervision, Funding acquisition, Conceptualization. **Hanhao Dai:** Writing – review & editing, Supervision, Funding acquisition, Conceptualization.

## Funding

This work was supported by National Natural Science Foundation of China (82472473), Joint Fund Project for Science and Technology Innovation (2021Y9023), Major Scientific Research Project of Fujian Province (2021ZD01003), Joint Project for Health and Education of Fujian Province (2019-WJ-01), Health Research Personnel Training Project of Fujian Provincial Health Commission (2019-CX-1), Science and Technology Planning Project of Fujian Province (2019J01173), Startup Fund for Scientific Research of Fujian Medical University (2020QH2050), Postdoctoral Science Foundation of China (2022M710702), Postdoctoral start-up fund of Fujian Provincial Hospital (0080132201), Fujian Provincial Natural Science Foundation Projects (2020J05270), Flint Fund project (2020HSJJ20), Fujian provincial health technology project (2020QNA009).

## Declaration of competing interest

The authors declare that they have no competing interests.

## Figures and Tables

**figs1:**
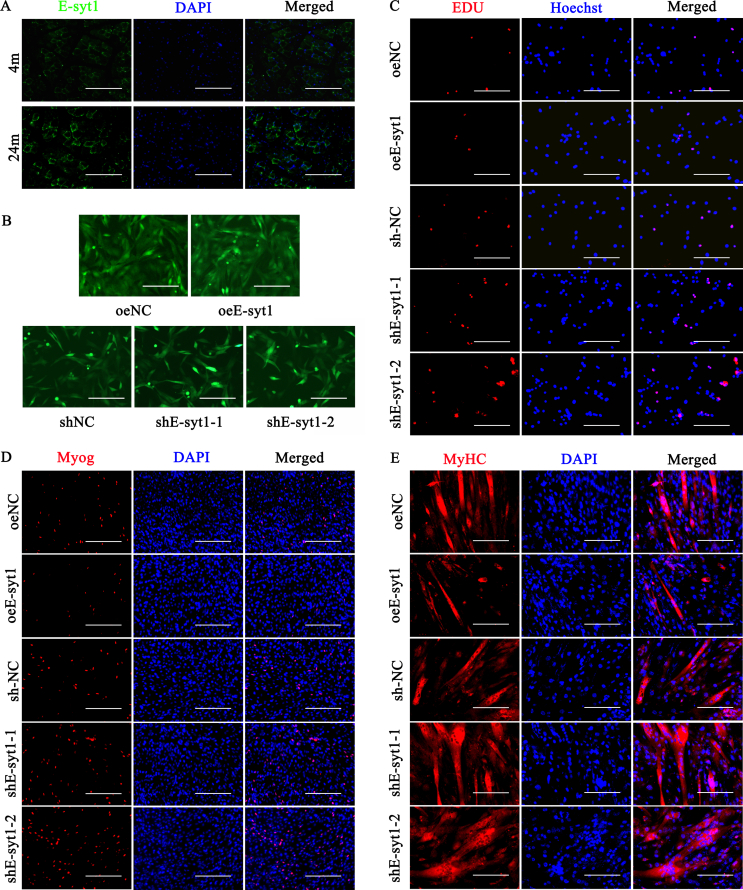


**figs2:**
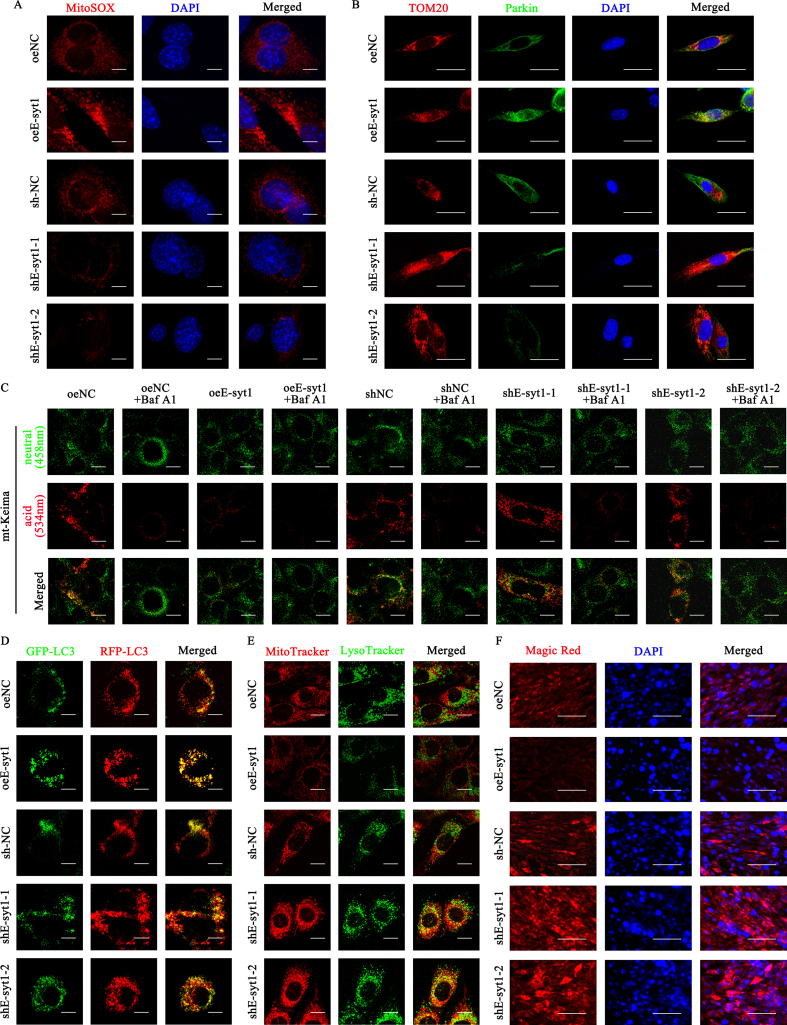


**figs3:**
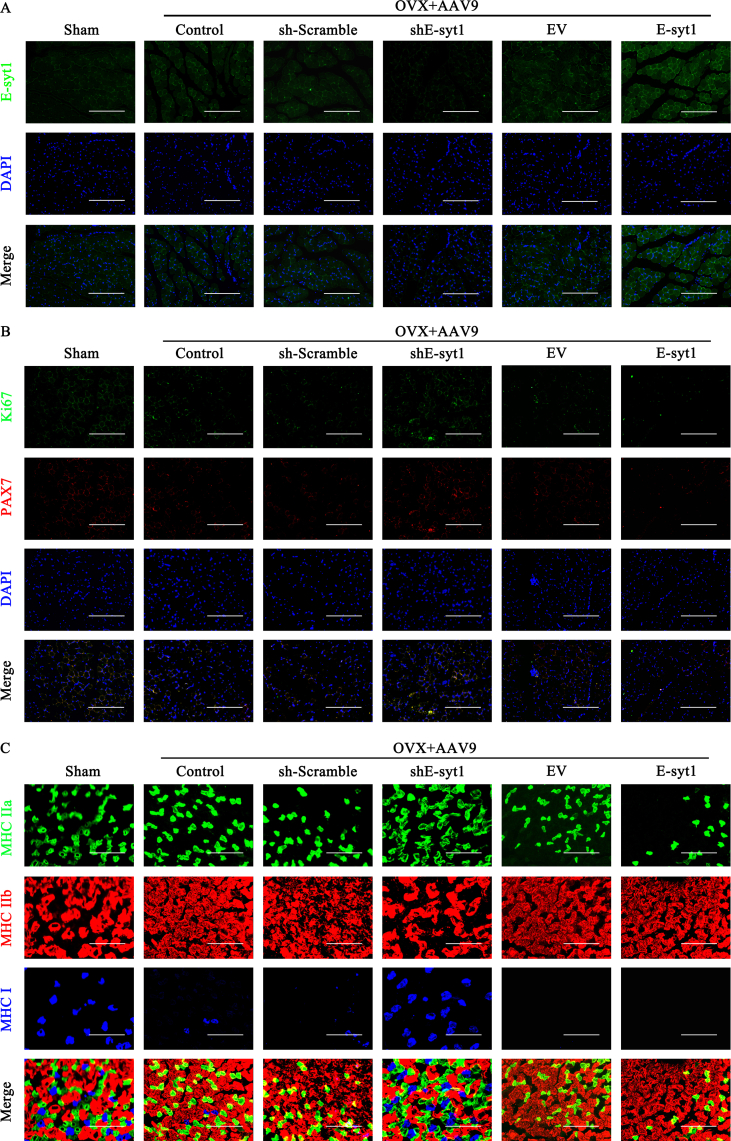


## Data Availability

Data will be made available on request.
