# Supplementary material for: Sarcopenic obesity is attenuated by E-syt1 inhibition via improving skeletal muscle mitochondrial function
Source: Redox Biol. 2024 Dec 12;79:103467. doi: 10.1016/j.redox.2024.103467 (PMC11699297; doi:10.1016/j.redox.2024.103467)
Supplement: Multimedia component 1 [file mmc1.docx]

**Supplementary Figure captions
Fig. S1.** E-syt1 inhibited proliferation and differentiation of C2C12 myoblasts. (A) Representative image of immunofluorescence staining of E-syt1. (B) At 48 h post transfection, fluorescence photographs of C2C12 cells transfected with oeNC, oeE-syt1, shNC, shE-syt1-1 and shE-syt1-2. (C) Representative EDU staining image in each group. (D, E) Representative image of immunofluorescence staining of Myog and MyHC in each group. (A -E) Scale bar = 200 μm.

**Fig. S2.** E-syt1 overexpression inhibits mitophagic flux, the fusion of mitophagosomes with lysosomes, and lysosomal activity. (A) Representative images of MitoSOX labeled mitochondrial ROS. (B) Representative images of mitochondria labeled with red (TOM20) and mitophagy labeled with green (Parkin) were utilized. (C) Representative mt-keima image of each group. (D) Representative GFP-LC3-RFP

image of each group. (E) Representative co-localization images of MitoTracker and LysoTracker in each group. (F) Representative Magic Red image of each group. (A, C-E) Scale bar = 10 μm. (B) Scale bar = 20 μm. (F) Scale bar = 200 μm.

**Fig. S3.** E-syt1 ablation improved mitochondrial homeostasis and increased the proportion of oxidative muscle fibers. (A) Representative image of immunofluorescence staining of E-syt1. (B) Representative co-localization images of PAX7 and Ki67. (C) Representative image of immunofluorescence staining of MHC I, IIa, IIb, and IIx myofibers. (A-C) Scale bar = 200 μm.
