# Supplementary material for: Sarcopenic obesity is attenuated by E-syt1 inhibition via improving skeletal muscle mitochondrial function
Source: Redox Biol. 2024 Dec 12;79:103467. doi: 10.1016/j.redox.2024.103467 (PMC11699297; doi:10.1016/j.redox.2024.103467)
Supplement: Multimedia component 2 [file mmc2.docx]

**Table S1 Primers used in RT-qPCR experiments**

| Name | Forward primer | Reverse primer |
| --- | --- | --- |
| E-syt1 | 5′-AGCGGTTTGAGTGGGATCTG-3′ | 5′-TCAGGTCATACCACTGGGCT-3′ |
| GAPDH | 5′-ATCTCTGCCCCCTCTGCTGA-3′ | 5′-GATGACCTTGCCCACAGCCT-3′ |
| Myostatin | 5′-TGGTCATGATCTTGCTGTAACC-3′ | 5′-CTTGACCTCTAAAAACGGATTCA-3′ |
| MuRF­1 | 5′-AGGGCTCCCCACCACTGTGT-3′ | 5′-TTGCCCCTCTCTAGGCCACCG-3′ |
| Atrogin-1 | 5′-ACTCTGCCAGTACCACTTCTC-3′ | 5′-CTGCTCTTTCCTTGGGTAACATC-3′ |
| Mito | 5′-CTAGAAACCCCGAAACCAAA-3′ | 5′-CCAGCTATCACCAAGCTCGT-3′ |
| B2M | 5′-ATGGGAAGCCGAACATACTG-3′ | 5′-CAGTCTCAGTGGGGGTGAAT-3′ |
