# Supplementary material for: Sarcopenic obesity is attenuated by E-syt1 inhibition via improving skeletal muscle mitochondrial function
Source: Redox Biol. 2024 Dec 12;79:103467. doi: 10.1016/j.redox.2024.103467 (PMC11699297; doi:10.1016/j.redox.2024.103467)
Supplement: Multimedia component 3 [file mmc3.docx]

**Table S2** **The sequences of shRNAs**

| Name | Sequence |
| --- | --- |
| shNC | 5′-TTCTCCGAACGTGTCACGT-3′ |
| shE-syt1-1 | 5′-TCACCGCAGAGACGCTTTATA-3′ |
| shE-syt1-2 | 5′-GACGTGGAGGTGAAGAAATAT-3′ |
| AAV9-sh-Scramble | 5′-CGCTGAGTACTTCGAAATGTC-3′ |
| AAV9-shE-syt1 | 5′-GACGTGGAGGTGAAGAAATAT-3′ |
