# Supplementary material for: Sarcopenic obesity is attenuated by E-syt1 inhibition via improving skeletal muscle mitochondrial function
Source: Redox Biol. 2024 Dec 12;79:103467. doi: 10.1016/j.redox.2024.103467 (PMC11699297; doi:10.1016/j.redox.2024.103467)
Supplement: Multimedia component 4 [file mmc4.docx]

| **Table S3 Antibodies and their application** | | | | | |
| --- | --- | --- | --- | --- | --- |
| Antibody name | Catalogue Number | Brand name | Dilution ratio | | |
|  |  |  | WB | IF | IP |
| Anti-GAPDH antibody | HRP-60004 | Proteintech | 1/10000 |  |  |
| Anti-Myod1 antibody | ab203383 | Abcam | 1/100 |  |  |
| Anti-Myog antibody | ab124800 | Abcam | 1/200 | 1/500 |  |
| Anti-MyHC antibody | ab91506 | Abcam | 1/1000 | 1/1000 |  |
| Anti-E-syt1 antibody | A15410 | ABclonal | 1/2000 | 1/200 |  |
| Anti-Ki67 antibody | A16919 | ABclonal | 1/1000 | 1/200 |  |
| Anti-MuRF­1 antibody | A3101 | ABclonal | 1/2000 |  |  |
| Anti-Atrogin-1 antibody | A3193 | ABclonal | 1/2000 |  |  |
| Anti-Myostatin antibody | A22725 | ABclonal | 1/2000 |  |  |
| Anti-β-actin antibody | 66009-1-Ig | Proteintech | 1/100000 |  |  |
| Anti-NDUFS1 antibody | 12444-1-AP | Proteintech | 1/10000 |  |  |
| Anti-SDHA antibody | A13852 | ABclonal | 1/2000 |  |  |
| Anti-UQCRC2 antibody | A4366 | ABclonal | 1/2000 |  |  |
| Anti-COX IV antibody | A6564 | ABclonal | 1/2000 |  |  |
| Anti-ATP5A1 antibody | A11217 | ABclonal | 1/2000 |  |  |
| Anti-LC3A/B antibody | #12741 | Cell Signaling | 1/1000 |  |  |
| Anti-p62 antibody | 66184-1-Ig | Proteintech | 1/5000 |  |  |
| Anti-VDAC1 antibody | A19707 | ABclonal | 1/2000 |  |  |
| Anti-PGC1α antibody | A12348 | ABclonal | 1/2000 |  |  |
| Anti-PGC1β antibody | A4331 | ABclonal | 1/2000 |  |  |
| Anti-Mfn1 antibody | A9880 | ABclonal | 1/2000 |  |  |
| Anti-Mfn2 antibody | A12771 | ABclonal | 1/2000 |  |  |
| Anti-Opa1 antibody | A9833 | ABclonal | 1/2000 |  |  |
| Anti-Drp1 antibody | A2586 | ABclonal | 1/2000 |  |  |
| Anti-Fis1 antibody | A19666 | ABclonal | 1/1000 |  |  |
| Anti-TOM20 antibody | sc-17764 | Santa Cruz |  | 1/50 |  |
| Anti-Pink1antibody | sc-517353 | Santa Cruz | 1/200 | 1/50 |  |
| Anti-Parkin antibody | sc-32282 | Santa Cruz | 1/200 |  |  |
| Anti-Flag antibody | #2368 | Cell Signaling | 1/10000 |  | 1/50 |
| Anti-Rabbit IgG(H+L) HRP | GAR007 | MultiSciences | 1/100000 |  |  |
| Anti-Mouse IgG(H+L) HRP | GAM007 | MultiSciences | 1/100000 |  |  |
| Anti-Pax7 antibody | bs-22741R | Bioss |  | 1/100 |  |
| Anti-Myh7 antibody | BA-D5 | DSHB |  | 1/6 |  |
| Anti-Myh2 antibody | SC-71 | DSHB |  | 1/12 |  |
| Anti-Myh1 antibody | 6H1 | DSHB |  | 1/4 |  |
| Anti-Rabbit IgG(H+L) (Alexa Fluor 594) | ab150084 | Abcam |  | 1/500 |  |
| Anti-Rabbit IgG(H+L) (Alexa Fluor 488) | ab150077 | Abcam |  | 1/500 |  |
| Anti-Mouse IgG(H+L) (Alexa Fluor 488) | ab150113 | Abcam |  | 1/500 |  |
| Anti-Mouse IgG2b, Alexa Fluor™ 488 | A-21141 | Invitrogen |  | 1/500 |  |
| Anti-Mouse IgG (H+L), Alexa Fluor™ Plus 405 | A48257 | Invitrogen |  | 1/500 |  |
| Anti-Mouse IgM, Alexa Fluor™ 555 | A-21426 | Invitrogen |  | 1/500 |  |
